# Effect of multi-refresh-rate method on user experience: sustained attention and inattentional blindness

**DOI:** 10.1186/s41235-025-00663-0

**Published:** 2025-08-15

**Authors:** Jieun Cho, Jeunghwan Choi, Cheongil Kim, Jeong Hyeon Park, Sang Chul Chong

**Affiliations:** 1 Graduate Program in Cognitive Science, Yonsei University, 50 Yonsei-ro, Seodaemun-gu, Seoul, 03722 Republic of Korea; 2 Center for Cognitive Science, Yonsei University, 50 Yonsei-ro, Seodaemun-gu, Seoul, 03722 Republic of Korea; 3 Department of Psychology, Yonsei University, 50 Yonsei-ro, Seodaemun-gu, Seoul, 03722 Republic of Korea

**Keywords:** Motion artifacts, Refresh rates, Display, Inattentional blindness, Attentional fluctuations

## Abstract

In today's digital world, understanding refresh-rate implications on visual perception and energy consumption is crucial. While high refresh rates enhance motion perception and user experience, they also increase energy usage, prompting the need for adaptive solutions like variable refresh rates. This study examines whether users notice or are affected by reduced refresh rates in task-irrelevant areas and examine whether variable refresh rates compromise a satisfactory display experience. Most participants failed to detect decreases in refresh rate in their peripheral view, and their task performance of the main task, which required sustained attention, remained unaffected. However, when informed of the possible change in the periphery, detection of it improved. In addition, during out-of-the-zone states, people with expectations about the phenomenon may be more likely to falsely report the change in the display. The findings suggest that centrally focused attention limits awareness of peripheral refresh-rate changes, supporting the potential of multi-refresh-rate strategies to optimize energy efficiency without compromising user experience.

**Supplementary Information:**

The online version contains supplementary material available at 10.1186/s41235-025-00663-0.

## Significance statement

How broadly does our spatial awareness extend on displays during active task engagement? The present study suggests that while attentively engaged in a task like watching a video in a split-window setup, individuals are less likely to notice an irregular visual phenomenon, such as motion juddering, in areas outside their primary focus. Furthermore, these unnoticed irregularities in peripheral displays do not significantly impact video-watching experience. This study provides vital strategies for developing energy-efficient and user-friendly displays in today's context of large, high-refresh-rate screens.

**Supplementary Information:**

The online version contains supplementary material available at 10.1186/s41235-025-00663-0.

## Introduction

The standards for display quality among users are continually rising. Users prefer large monitors and mobile phone screens, which enable them to divide these large displays into multiple windows and switch between tasks. For example, someone can text a friend while watching an ice hockey game on a mobile phone using the split-view function. Furthermore, users increasingly seek high-refresh-rate displays for improved visual experiences (e.g., smooth movements of hockey players and pucks), as higher refresh rates reduce visual artifacts like flickering and motion blurring. These artifacts not only degrade the perceived quality of visual content but can also cause discomfort (Davis et al., 2015; Denes et al., 2020; Landis, 1954). Additionally, lower refresh rates can negatively impact visual performance. Studies show that people react more slowly to color changes at 60 Hz compared to 240 Hz (Murakami et al., 2021). Similarly, tasks like speed discrimination and reading are performed better at a refresh rate of 1,000 Hz than at 60 Hz (Kime et al., 2016). As these findings highlight, there is a growing demand for displays that support higher refresh rates. The most common refresh rate for consumer displays, 60 Hz, is utilized in televisions and computer monitors. However, smartphones and tablets typically support 90 to 120 Hz, and gaming displays often exceed 144 Hz. These values provide a practical baseline for designing refresh-rate conditions and understanding user experiences.

Despite the benefits of high-refresh-rate displays, they consume significantly more energy, especially on large screens, and are particularly problematic for mobile devices where battery life is critical. High refresh rates drain battery life faster than lower refresh rates, leading to the increasing trend of using adaptive or multi-refresh-rate displays to conserve energy (Kim et al., 2007; Kim et al., 2021; Kim et al., 2022; Nixon et al., 2014; Park & Kim, 2019; You et al., 2020). This trend raises an important question: How can we extend battery life and save energy without diminishing the user experience?

To address this, one solution is to implement adaptive refresh rates, where different areas of the screen refresh at different rates depending on the content. For example, peripheral dimming can be used by tracking a user’s gaze, based on the understanding that peripheral vision is less precise than foveal vision, so lowering the refresh rate or dimming the periphery might not negatively impact user experience (Kim & Lee, 2020). Similarly, a multi-frequency method has been proposed, where refresh rates are kept high for video content but lowered for still images, which reduces power consumption without significantly affecting the viewing experience (Kwon et al., 2022). However, these studies did not focus on whether users notice or are affected by changes in peripheral areas of the display, which could be critical for evaluating multi-refresh-rate displays. This is particularly relevant when users are managing multiple windows, as a peripheral window may have a lower refresh rate than the main window where the user’s attention is focused.

This study builds on the knowledge of human peripheral vision and attention to investigate whether users notice a drop in refresh rates in a secondary window placed in the periphery. It also examines whether this decrease impacts performance in the main window, which is located around the foveal area. Research on peripheral perception shows that our awareness of the periphery is more limited than we might assume. For example, Cohen et al. (2020) found that participants exploring a virtual environment without a specific task often failed to notice color changes in their peripheral vision. This suggests that we may not be aware of certain visual changes in the periphery when our attention is focused elsewhere. In a multi-window display setup, if we concentrate on one window, we might not notice any visual issues, such as motion artifacts in another, peripheral window. Thus, reducing the refresh rate in peripheral windows could be a viable energy-saving strategy without affecting the user's main task.

On the other hand, it is also possible that motion-related artifacts, like those caused by lower refresh rates, are easily noticed in the periphery. Previous studies have shown that motion artifacts are more easily perceived in the peripheral areas of vision (Krajancich et al., 2021). In fact, high-frequency luminance flicker, which may not be noticeable in central vision, can be detected in the periphery (Solomon et al., 2002; Tyler, 1985; Waldin et al., 2017). Peripheral vision is particularly sensitive to motion (Lappin et al., 2009), and even when people are focused on a central task, they can detect motion in the periphery during dual-task situations (Vater et al., 2017).

Moreover, attention fluctuates over time, and our focus can shift even when we are concentrating on a specific area. These shifts in attention can affect our ability to detect events in the periphery. During periods when attention shifts, it may become more likely to notice motion artifacts in the peripheral window, even if they do not initially capture attention (Esterman & Rothlein, 2019; Fiebelkorn & Kastner, 2019). If this is the case, reducing the refresh rate of a secondary window may not be a good solution, as users might still notice the lower quality and be distracted.

To test these possibilities, this study examined whether participants could detect a reduction in the refresh rate of a window located in the periphery. The experiment was conducted in two sessions. In the first session, participants were instructed to focus on a main task in a central window while ignoring a peripheral window. The main task was a sustained attention task, known as the gradual-onset continuous performance task (gradCPT; Esterman et al., 2013; Jun et al., 2019), designed to measure fluctuations in attention over time. This task required participants to sustain their attention for 8 minutes, during which they had to report specific scene categories in the task-relevant window.

The peripheral window contained a simple video of a red dot rotating around a circle, similar to the clock task used by Libet (Lau et al., 2004; Libet, 1999). At a random point during the task, the refresh rate of the peripheral window was reduced for 30 seconds. After the first session, participants were asked whether they noticed any changes in the motion of the red dot. They were then shown the actual refresh rate changes that had occurred during the task. In the second session, participants were asked to repeat the task but to report any detection of refresh rate changes immediately upon noticing them. This allowed us to assess whether participants could detect the changes when they were paying attention to the peripheral window and when memory decay was not a factor.

## Method

### Participants

A total of 80 undergraduate and graduate students (55 women, 25 men; age range $=$ 19–32 years, mean = 21.84 years) were recruited from Yonsei University in 2023. They were randomly assigned to either the 20 Hz refresh rate group or the 30 Hz refresh rate group, which resulted in 40 participants in each group. The initial number of participants per group was determined as the minimal sample size following Cohen et al. (2021), which used an inattentional blindness paradigm to assess individuals’ lack of awareness regarding peripheral visual experiences. In their study, the authors parametrized the extent of this target phenomenon across different groups, each consisting of 40 participants. Following an optional stopping rule, we first stopped data collection after this minimal number of participants and performed data analysis using the Bayesian method (Rouder, 2014; Schönbrodt & Wagenmaker, 2018; Schönbrodt et al., 2017). Participants received either 1 course credit or 6,000 won for their participation. All participants reported normal or corrected-to-normal visual acuity. The experimental protocol was approved by the Institutional Review Board at Yonsei University, and written informed consent was obtained according to the approved procedures.

### Apparatus and stimuli

#### Apparatus

Stimulus presentation, response recording and data collection functions were performed using MATLAB 2023a (MathWorks Inc., Natick, MA, USA) in conjunction with Psychophysics Toolbox 3 (Brainard, 1997; Pelli, 1997). The stimuli were displayed on an LCD monitor at a resolution of 1,920 × 1,080 with a refresh rate of 360 Hz. Viewing distance was not strictly constrained but was approximately 1 m. Some participants who reported recognizing strange phenomena during our inattentional blindness questioning phase used a laptop computer to type their responses freely without any constraint.

#### Stimuli

**Figure 1:**
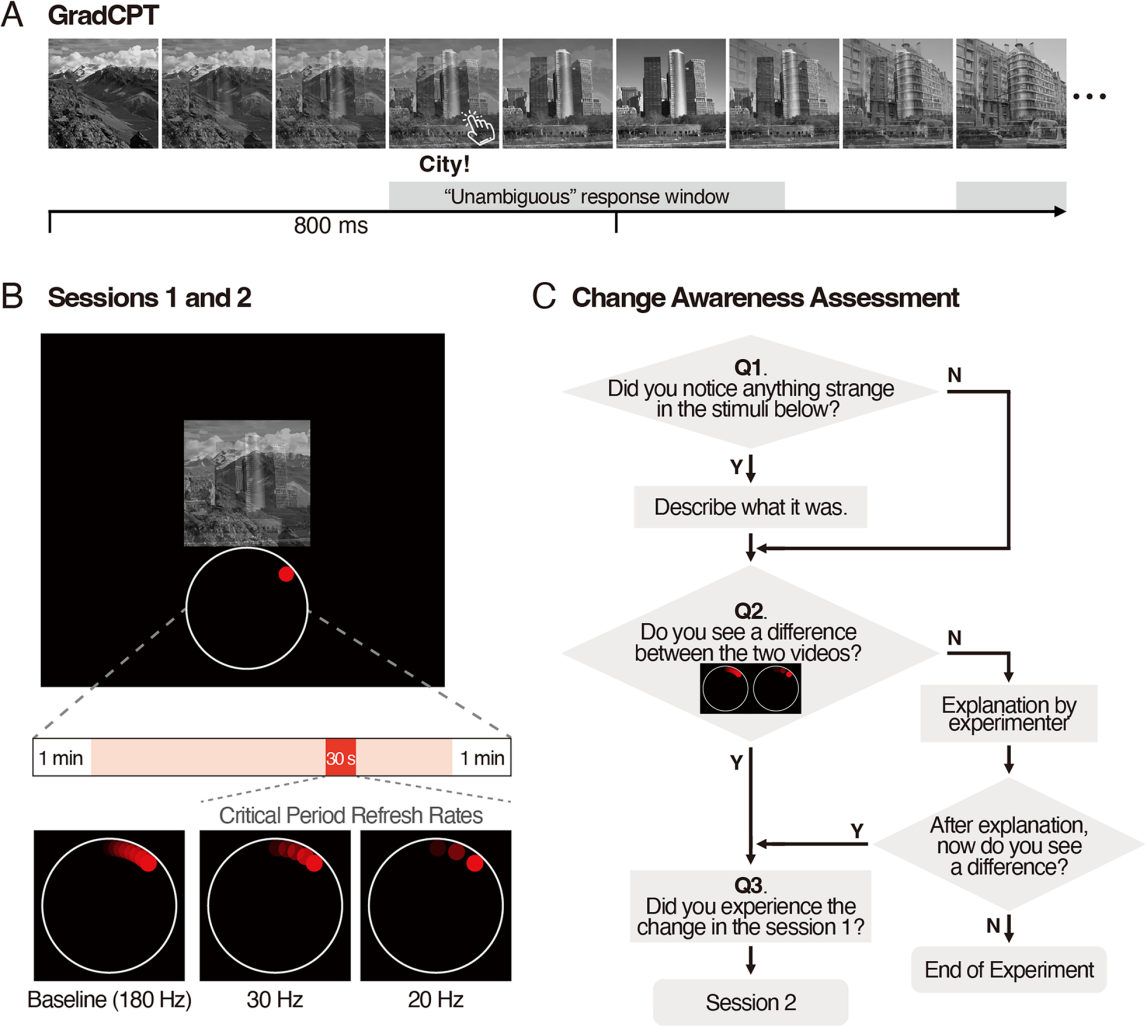
Illustration of gradCPT, the Task Window and Question Sequence. **A** An example sequence of scenes in the gradCPT task. In this example, participants are instructed to respond to city images by pressing the “c” key and to withhold responses to mountain images. The ticks in the horizontal line schematically indicate the start and the duration of each trial. The grey rectangular boxes mark the time windows during which participants’ responses can be categorized as unambiguous. **B** A demonstration of the task window. A continuous stream of scenes was presented at the center of the display, with a video of revolving red dot below the scene display. The refresh rate was reduced for 30 s (indicated by a red rectangle in a bar) at a random time, from a baseline to one of the frequency conditions, depending on the group (either 30 Hz or 20 Hz). **C** Flowchart of the questions posed to participants after the first session.

In the sustained attention task, we used 10 mountain and 10 city-scene images. These images were centrally presented on the display, with a size of 4.14˚ $\times$ 4.14˚ of the visual angle (Figure 1A). In each trial, a scene image was gradually transformed into a different scene through linear pixel-by-pixel interpolation. The sequence of the presented stimuli and the combination of the two scenes per trial were randomly determined for each participant.

Below the sustained attention task window, we presented a video of a small red dot moving around a large white circle (Fig. 1B). The radius of the white circle was 2.02˚ in visual angle, and the radius of the small red dot was 0.16˚ in visual angle.

#### Design and procedure

The experiment consisted of two 8-minute sustained attention task sessions with an in-between change awareness assessment session to investigate whether participants had been aware of the occurrence of low-refresh-rate moments during the sustained attention task (Fig. 1B). In the beginning, participants performed practice trials for 30 s to become familiar with the gradCPT (Esterman et al., 2013; Jun et al., 2019; Rosenberg et al., 2013). They were asked to respond upon seeing target scenes and withhold responses to nontarget scenes while viewing a rapidly changing and continuous stream of scenes. For half the participants, the target scenes were city images and the nontarget scenes were mountain images, and for the other half, the condition was reversed, i.e., the target and nontarget scenes were mountain and city images, respectively. The participants were asked to ignore stimuli presented below the main window and solely focus on the main task.

The 8-minute gradCPT session consisted of 600 trials (540 target and 60 nontarget trials). In each trial, a scene to detect gradually faded in and then faded out in the next trial, each lasting 800 ms (Fig. 1A). This effect was achieved by linearly interpolating the pixel values of temporally adjacent trials' scene images. The weight of the scene image for trial n gradually increased from 0% at the beginning of the trial to 100% at the start of trial n+1. Subsequently, it gradually decreased to 0% by the end of trial n+1. This procedure is identical to previous studies that have adopted gradCPT (Esterman et al., 2013; Jun et al., 2019). The weights of the two adjacent scenes were adjusted every 25 ms, creating a seamless blend of scenes through 32-step gradual changes per trial. The adjustment interval was set to half that of the previous study (50 ms; Jun et al., 2019) to reduce the sense of flicker reported during the pilot study.

During the continuous transition of scenes in the main task area, the red dot moved at a baseline refresh rate of 180 Hz. The red dot shifted by one degree every 5.56 ms, resulting in a complete circle rotation lasting 2 s (Fig. 1B). This rotation time remained consistent even when transitioning to low refresh rates. At a random time point, excluding the first and last minute of a trial, a degradation in the refresh rates started to occur, affecting the way that the red dot revolved. This was defined as the critical period. The refresh rates decreased in gradual steps, from the baseline of 180 Hz to 90 Hz, 60 Hz, 45 Hz, 36 Hz and, finally, to the target refresh rate of either 30 Hz or 20 Hz. During each step, the red dot revolved half a cycle within a second. Upon reaching the target refresh rate, the red dot revolved for 22 s, after which it gradually returned to the baseline refresh rate over a span of 4 s, following the inverse sequence of steps (36 Hz → 45 Hz → 60 Hz → 90 Hz → 180 Hz). These gradual decrements and increments were implemented to avoid abrupt refresh-rate transitions, which could increase visual saliency and attract attention, potentially disrupting the measurement of inattentional blindness. Supporting this approach, Han et al. (2023) demonstrated that gradual transitions between flicker frequencies significantly reduced perceptual sensitivity to temporal changes. We chose target refresh rates of 30 and 20 Hz for the following reasons. First, we wanted them to be below the typical flicker fusion threshold of 50 Hz (Cai et al., 2024; Burr, 1991; see also Denes et al., 2020; Dovale, 2017 for critical refresh rate threshold). Eisen-Enosh et al. (2017) measured flicker fusion thresholds across a range of refresh rates from 10 to 60 Hz and determined that the thresholds were approximately between 30 and 40 Hz. Second, with the advent of adaptive refresh rate technologies, some devices are capable of reducing their refresh rates to as low as 10 Hz during low-demand activities such as reading emails, browsing the web, or texting (Samsung Newsroom, 2020). Therefore, within the threshold limit, we wanted to reduce refresh rates as low as possible to maximize energy saving.

Immediately after completing the first gradCPT session, the participants were subjected to a series of inquiries that assessed their awareness of the change in the refresh rates during their task (Fig. 1C). First, we asked whether they had experienced anything strange in the stimulus positioned below the main task stimuli. We asked the participants to recall their experience with minimum guidance to avoid provoking any bias in their recall. If they responded “Yes” to this question, to check whether their response to the first question was based on the exact experience that we targeted rather than a random guess, they were asked to provide a description by typing it on a laptop computer. For those who replied “Yes” or “No” to the first question, a second question followed. In the second question, participants observed two videos of red dots moving that were concurrently displayed: one on the left and the other on the right. They were asked whether they could perceive any perceptual differences between the demonstration videos (baseline refresh rate video vs. 20/30 Hz video). The participants who provided a positive answer proceeded to the third question. However, for those who responded with a negative answer, the experimenter offered an explanation:

*“The red dot in the left (or right) image moves by 1 degree, whereas the dot in the right (or left) image moves by 9 degrees (or 6 degrees). Consequently, the movement of the dot on the left (or right) seems to be less continuous or more distorted in comparison to the other.”*

Following the explanation, participants were asked again whether they now perceived the difference. If they continued to reply negatively, they did not proceed to the second session because their failure to detect the change might have been a perceptual rather than attentional deficit. Before proceeding to the third question, the participants who acknowledged the perceptual difference were informed about the red dot’s rotation at a low refresh rate for a brief period during the first session.

Given that the participants were aware of and understood the manipulation in the critical period, the third question asked them to recall whether they had encountered the change demonstrated and explained by the experimenter during the first session. The purpose of the third question was to investigate whether the participants could better recall their recent experiences using the provided demonstration and explanation of the manipulated phenomenon and to compare the responses with those of the first question, where participants may have doubted their perception of the phenomenon due to uncertainty or a lack of confidence given the minimal information provided when initially questioned. Following their responses, participants proceeded to the second session, which was an additional 8-minute gradCPT task. Here, participants were given explicit instructions concerning the potential occurrence of the previously explained critical period where the refresh rate decreases at a random moment. They were asked to mainly focus on the sustained attention task as in the first session. However, upon observing the phenomenon, participants were instructed to press the space bar to report their detection and then promptly resume the main task.

#### Data analysis

**Accuracy and Response-Time Variability Analysis of gradCPT.** To analyze accuracy and response times (RTs) during the sustained attention task, we first adopted the response classification procedure of previous gradCPT studies (e.g., Esterman et al., 2013; Jun & Lee, 2021; Jun et al., 2019; Rosenberg et al., 2013), with slight changes in the parameters. First, we classified each trial as unambiguous for those responses made when the scene image was a 60%/40% morph between the current and previous trials (480 ms after the onset of the current trial) and before it became a 70%/30% morph between the current and next trials (240 ms after the onset of the next trial) (Fig. 1A). Trials for which responses were made outside of this period were initially classified as ambiguous. Our coherence range for categorizing a trial as unambiguous was shifted 80 ms earlier than that of original gradCPT studies, while keeping its total duration unchanged at 560 ms. This modification was made to reduce the proportion of ambiguous trials—which reached 29% under the original method—and to better align with the range reported in previous studies (up to 18%; Jun et al., 2019). Notably, slight variations in the parameters that affected the categorization of each trial did not change the patterns of the results reported below.

In the first and second sessions, 13.87% and 18.8% of the trials were categorized as ambiguous, respectively. These ambiguous trials were analyzed in the following steps. First, they were assigned to adjacent trials that had no responses. If neither adjacent trial had a response, the response was assigned to the go trial. However, if both trials were either go or no-go trials, responses shorter than or equal to 480 ms were assigned to the previous trial, while responses longer than 480 ms were assigned to the current trial. Following previous conventions (e.g., Rosenberg et al., 2013), we also did not use 400 ms as the reference point, which was the half point of one trial, to consider the time taken to make a decision.

To measure within-subject attentional stability and the level of attentiveness during the sustained attention task, we analyzed the variance time course (VTC) of each participant’s RTs, following a previously established method (Esterman et al., 2013). This analysis is grounded in the assumption that substantial fluctuations in RTs over a period reflect a diminished attentional state (out-of-the-zone epoch). This is because not only can extremely slow RTs often indicate reduced attention, resulting in an extended time to determine scene category from the ongoing stream of images, but extremely fast RTs can also indicate an inability to inhibit responses or responses made without full attention. Conversely, a consistent and low variability in RTs indicates a heightened attentive state (in-the-zone epoch).

The VTC was computed by using only correct response trials of the gradCPT task. Incorrect and no-response trials were interpolated using the RTs of the 2 surrounding trials. The VTC was smoothed using a Gaussian kernel of 9 trials full width at half maximum, which integrated information from the surrounding 20 trials. By splitting the smoothed VTC with a median value, we divided periods into low-variability (in-the-zone) or high-variability (out-of-the-zone) epochs.

**Coding Criteria for Correct Change Detection in Second Session.** In the second session, we asked the participants to respond when they observed the degradation of refresh rates manifested in the revolution of the red dot. We instructed the participants to press the space bar once they detected the degradation and then promptly resume the main task. However, we found that participants showed different numbers of detection presses ranging from 0 to 42. The presses occurred in both the non-critical period (when the red dot revolved at a baseline refresh rate) and the critical period (when the refresh rate was degraded to either 20 Hz or 30 Hz). We labeled presses as false alarms when participants pressed a space bar outside the critical period and as hits when participants pressed it during the critical period. Considering the varying number of presses among participants, we evaluated the accuracy of each group's ability to detect the degradation during the critical period using liberal and conservative criteria. Under the liberal criterion, a participant was considered to have responded correctly if they pressed the button at least once during the critical period. On the other hand, the conservative criterion required that a participant's every press should occur exclusively during the critical period for them to be categorized as a correctly responding participant. We report the percentage of participants classified as correctly responding according to both criteria to provide the overall range of accuracy.

**Excluded Data.** When analyzing the first session data, we used all 80 participants. When analyzing the second session data, we used only 75 participants, because four participants did not perform the second session as they could not perceptually discriminate our manipulated phenomenon and one participant did not respond at all for the sustained attention task. Other than this, we neither excluded nor trimmed specific trials.

**Statistics.** In this study, we employed a Bayesian approach to analyze and interpret the data. First, we examined potential differences in awareness of the critical period across different refresh-rate groups. To address this, we conducted independence testing between the two groups using a Bayesian analysis of contingency table with the BayesFactor package in R. The analysis was conducted with default priors and an independent multinomial sampling scheme as outlined in Jamil et al. (2017). Second, we conducted a Bayesian one-sample *t*-test to examine the relationship between individual participants’ attentional states and their presses.

When analyzing gradCPT performance, full models consisted of predictors and a participant-level random intercept, and null models contained only the random intercept. We used the brms package to conduct Bayesian mixed-model analysis (Bürkner, 2017) and the bayestestR package to compare models (Makowski et al., 2019). The models were fitted using eight chains, each with 6000 iterations, including 1000 warm-up samples per chain. This resulted in 40,000 Markov chain Monte Carlo samples, which is recommended for precise Bayes factor calculation. For the population-level effects in the model, we used Student’s *t* distribution (ν = 4, µ = 0, s = 2.5) as the prior, which is a weakly informative prior. For the intercepts, we chose a Cauchy distribution (0, 10) as the prior, which is also a weakly informative prior. We analyzed the effect of each predictor by calculating the inclusion Bayes factors (BF_incl_) across matched models (van den Bergh et al., 2020). For the Bayesian *t*-test analyses, we used the BayesFactor package with a standard Cauchy prior (Rouder et al., 2009).

Overall, we report the Bayes factors (BF_10_) to explain the results, which indicate the extent to which the data are better explained by one model than another (Hinne et al., 2020). For example, a BF_10_ of 3 indicates that the observed data are three times more probable under the alternative model, *H*_*1*_, than the null model, *H*_*0*_. As established in the previous literature, we define 1 < BF_10_ < 3 as anecdotal evidence, 3 < BF_10_ < 10 as moderate evidence, 10 < BF_10_ < 30 as strong evidence and BF_10_ > 30 as very strong evidence (Schönbrodt et al., 2017).

## Results

### Awareness of refresh-rate decrement

**Figure 2:**
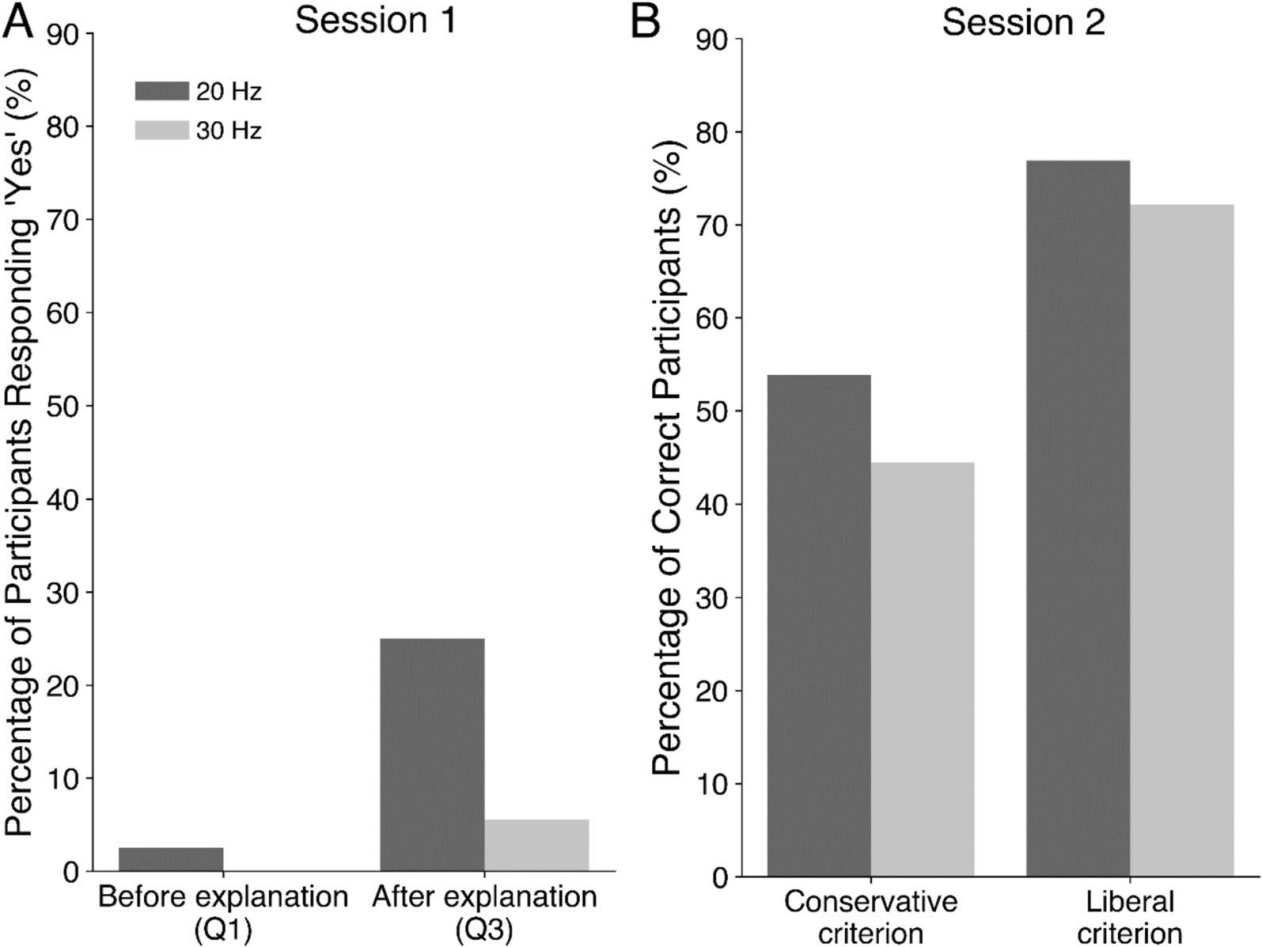
Awareness Results for Each Session. **A** Percentage of participants who responded “Yes” to the post-session questionnaires before the experimenter’s explanation about the critical period (Q1: “Did you notice anything strange?”) and after (Q3: “Did you experience the change in the session 1?”), respectively. We excluded participants who provided incorrect written descriptions despite their “Yes” responses to Q1. **B** The percentage of correct participants according to the two criteria: liberal and conservative.

In the first session, our objective was to assess participants’ awareness of refresh-rate changes while engaged in an attention-demanding task. In the first question after the first session, only 10% of the participants (four people in each group) reported observing oddities in the task-unrelated window and described them in their written responses. Our analysis of the descriptive responses revealed that only one participant in the 20 Hz group provided a response that closely matched our experimental manipulation. (*“The image was presented with a slight time delay, causing it to appear overlapped and fused,*” see Supplementary Table 1 for other responses.) By considering only the qualitatively accurate responses (Fig. 2A), we found moderate evidence in favor of the null hypothesis suggesting no significant difference between the two groups in terms of the awareness of the change in refresh rates (BF_10_ = 0.17).

For the second question, we confirmed a stark perceptual difference between the two refresh-rate groups and that the lack of awareness was not due to the perceptual subtlety of the changes in the refresh rates. Participants first viewed two videos simultaneously displaying a rotating circle at a baseline refresh rate (180 Hz) and the target refresh rate (30 Hz or 20 Hz) and then were asked about the perceptual difference between the two videos. All participants in the 20 Hz group reported that they could distinguish the changed-refresh-rate video from the baseline, whereas 57.5% in the 30 Hz group reported observing a perceptual difference between the videos. This difference between the two groups was supported by very strong evidence of Bayes factors (BF_10_ = 55113.43). However, after receiving a detailed explanation of the manipulation, 90% in the 30 Hz group reported that they were able to recognize the perceptual difference. Regarding the third question where we re-asked about the critical period, 5.5% of the participants in the 30 Hz group and 25% of the participants in the 20 Hz group provided a positive answer. Independence testing between the two groups showed anecdotal evidence (BF_10_ $=$ 2.95).

In the second session, we investigated whether an explicit instruction to detect the change would increase the participants’ detection rate. We calculated the rate in two different ways by either applying the liberal or conservative criteria to determine whether a participant had correctly responded in the critical period. When applying the liberal criteria, the detection rate was 72.22% in the 30 Hz group and 76.92% in the 20 Hz group. When applying the conservative criteria, the detection rate was 44.44% in the 30 Hz group and 53.85% in the 20 Hz group (Fig. 2B). This range of detection rates was higher than that of the first session, indicating that expectation played an important role in detecting the degradation of the refresh rate. There was no evidence of group differences (BF_10_ $=$ 0.27 and BF_10_ $=$ 0.38 for the liberal and conservative criteria respectively), irrespective of the coding criteria.

### Effect of refresh-rate decrement on sustained attention task performance

Next, we analyzed whether the critical period affected participants’ sustained attention task performance despite their lack of explicit awareness about the degradation. The Bayesian *t*-test analysis revealed moderate evidence toward the null hypothesis that there was no difference in sustained attention task performance between the critical (mean accuracy = 90.47, *SD* = 6.95) and non-critical periods (mean accuracy = 90.41, *SD* = 5.19) in the first session (BF_10_ = 0.12). Moreover, we evaluated whether the critical period affected participants’ attention state measured with RT variability analysis. We calculated the proportion of in-the-zone moments during the 30 seconds of the critical period and tested whether this differed from 50%, the participants’ overall in-the-zone proportion for the entire session. The Bayesian *t*-test analysis found moderate evidence toward the null hypothesis that the in-the-zone proportion was not different from 50% (BF_10_ = 0.16). Altogether, these results suggest that when the participants did not have any expectation of the phenomenon, they were largely unaware of the degradation in the refresh rate, and the degradation did not affect participants’ performance in the main task.

### Effect of instructions to detect refresh-rate decrements on sustained attention task performance

**Figure 3:**
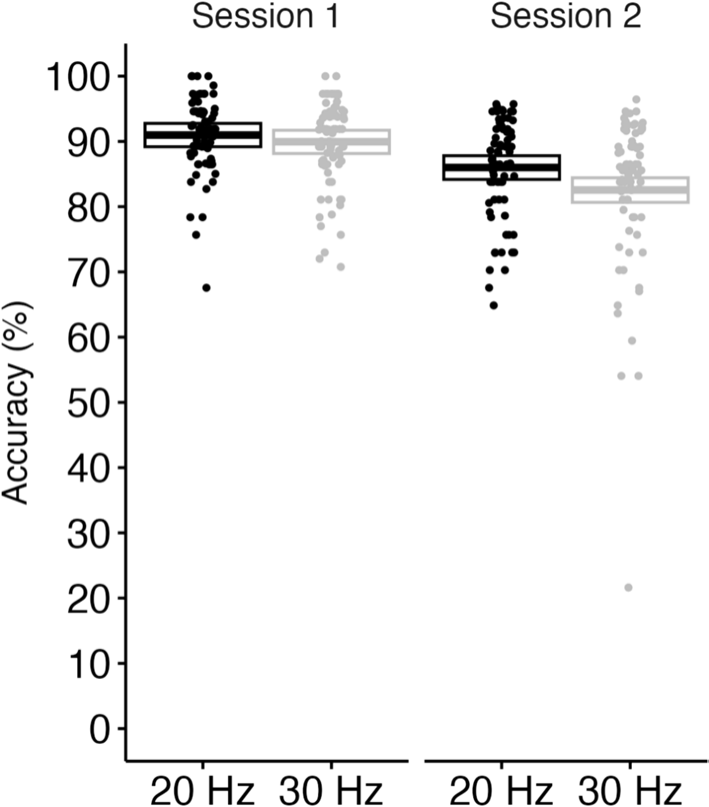
Accuracy of gradCPT. Central marks of box plots correspond to the estimate; the upper and the lower hinges indicate the lower and upper estimates.

We analyzed gradCPT performance across two sessions to assess how instructions to detect refresh-rate decrements influenced performance on a sustained attention task. As displayed in Fig. 3, we found strong evidence of interaction between the session and the group (BF_10_ = 11.01). There was also strong evidence of group (BF_10_ = 38.46) and very strong evidence of session (BF_10_ = 2.02e+09). These results suggest that the dual task of detecting the change in the refresh rate deteriorated participants’ performance of the sustained attention task, especially when the changes were subtle and less detectable at 30 Hz.

### Effect of attentional states on sustained attention task performance

**Figure 4:**
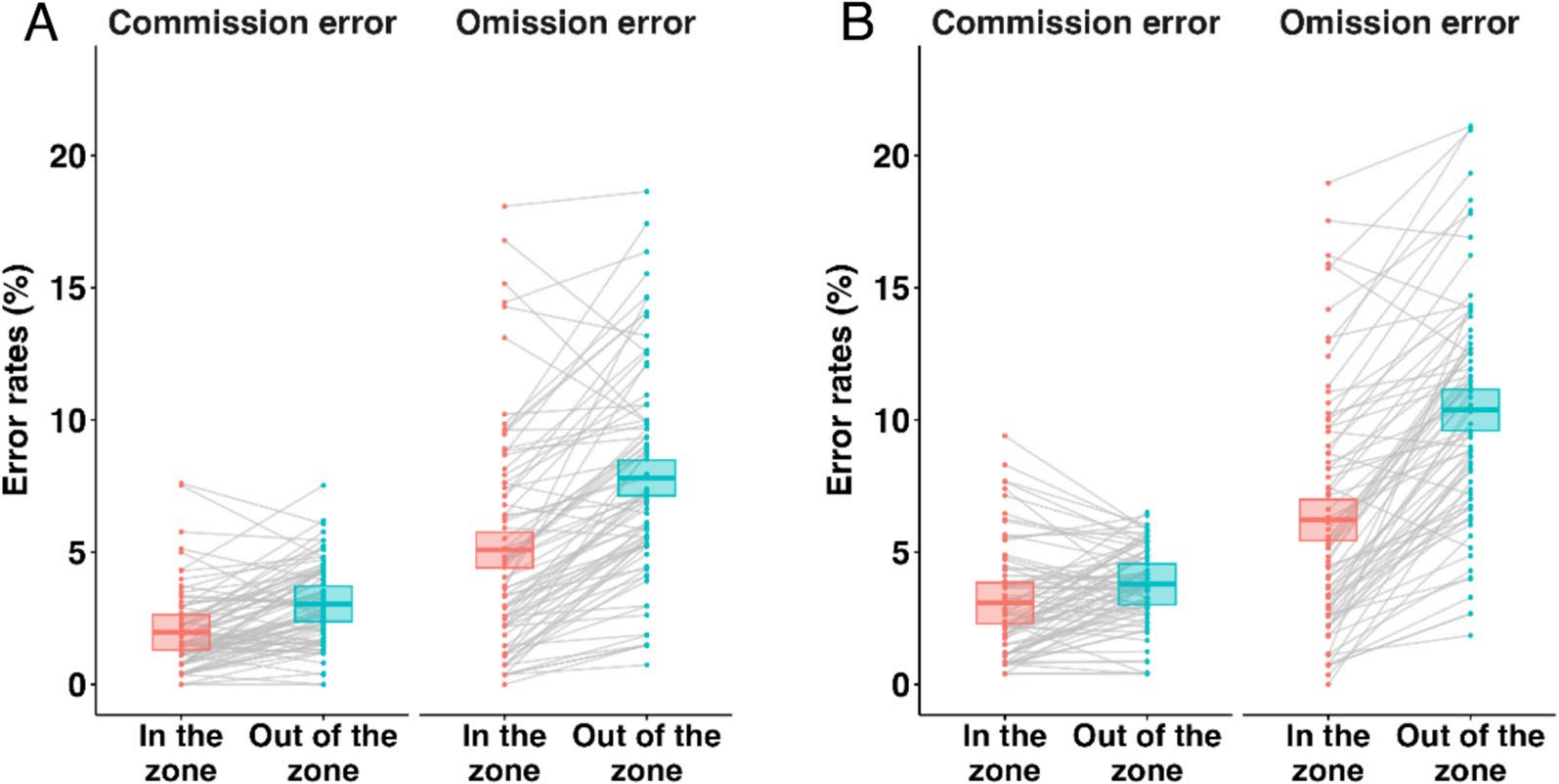
Error Rates by Error Types and Attention State. **A** Results of the first session. **B** Results of the second session. The boxplots consist of the estimates and the lower and the upper estimates.

We analyzed the performances according to two types of errors—commission error (proportion of no-go trials where participants made a response) and omission error (proportion of go trials where participants failed to respond)—during the in-the-zone and out-of-the-zone states, respectively (Fig. 4). In both sessions, participants made more omission errors than commission errors, as corroborated by very strong evidence of error type (BF_10_ = 3.45e+28 for the first session and BF_10_ = 2.00e+28 for the second session). Participants also made more errors in the out-of-the-zone versus the in-the-zone-state trials in both sessions, which is corroborated by very strong evidence of attention state (BF_10_ = 1.21e+08 for the first session and BF_10_ = 1.82e+08 for the second session) and replicates the error tendency found in previous gradCPT studies (Esterman et al., 2013). Lastly, we found very strong evidence of interaction between the two factors in both the first (BF_10_ = 66.56) and second sessions (BF_10_ = 1.56e+05). These results suggest that participants made more errors during the out-of-the-zone than in-the-zone states, and this tendency appears stronger for omission errors than commission errors.

### Effect of attentional states on detection of refresh-rate decrements

**Figure 5:**
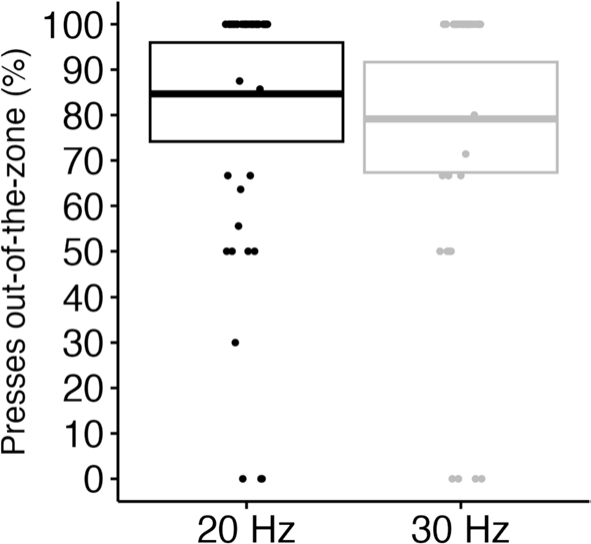
Proportion of Pressed Trials during the Out-of-the-zone. Central marks of box plots correspond to the estimate; the upper and the lower hinges indicate the lower and upper estimates.

We determined whether there is a relationship between the participants’ attentional state and the detection of refresh-rate changes in the second session (Fig. 5). Because many participants unexpectedly responded more than once for detecting the critical period, we were not able to create a model to examine whether a participant’s attention state predicted whether they correctly recognized flickering. Instead, factoring in all kinds of responses including false alarms, we examined in which attentional state (i.e., in-the-zone or out-of-the-zone) participants made more responses that indicated their detection of refresh-rate changes. Considering that the mean number of keyboard presses was variable among the participants, we calculated the proportion of out-of-the-zone presses compared to all presses. We excluded two participants who made no response in the critical period.

The Bayesian *t*-test analysis revealed that the proportion of out-of-the-zone responses was significantly higher than 50% in both the 20 Hz (BF_10_ = 1.78e+16) and 30 Hz groups (BF_10_ = 7.08e+07). There was also very strong evidence that the proportion of responses was higher in the 20 Hz group than in the 30 Hz group (BF_10_ = 54.43) (Fig. 5). Even when we excluded the trials during the critical period, this tendency was maintained. These results indicate that, in general, participants were more likely to press a key to report flickering in an out-of-the-zone moment than in an in-the-zone moment. Moreover, the difference between the 20 Hz and 30 Hz groups indicates that in the 20 Hz group, participants were more perceptive and attentive to the peripheral window than the 30 Hz group, even in an in-the-zone state.

## Discussion

Our increasing reliance on digital displays for activities such as gaming, reading and watching has driven advancements in display technology, notably toward higher refresh rates and larger screens. While these enhancements aim to provide more vivid and immersive digital experiences, this pursuit of high-end functionality has its disadvantages. For example, enhanced visual quality often leads to increased energy consumption, potentially reducing the overall usage time of these devices. Furthermore, the heightened sensory stimulation from these advanced displays can contribute to user fatigue. Balancing these factors is crucial for sustainable and user-friendly digital technology developments.

This study investigates whether people detect refresh-rate decrements in a peripheral secondary window and how these decrements impact performance on a primary task in a foveal main window. Overall, our findings suggest that people were unaware of an unexpected refresh-rate decrement in a secondary window while completing a primary task in a main window, and the refresh-rate decrement did not affect their performance on the primary task. In the first session, nearly all the participants (79 out of 80) failed to recognize this decrement until they were presented with a demonstration and received explanations about the critical period in the secondary window. Even after the detailed explanation of the refresh-rate decrement, there was only a marginal increase in the percentage of participants who responded “Yes.” This suggests that participants’ “No” responses to the first question (“Did you notice anything strange in the stimuli below?”) might not have been due to conservative response biases stemming from uncertainty or lack of confidence about the meaning of “anything strange.”

In the second session, more participants noticed the event than in the first session, even when applying a conservative coding accuracy criterion. This result suggests that anticipation of the refresh-rate decrement or anticipation-based spatial attention toward the secondary window played an important role in detecting the refresh-rate decrement and that the initial unawareness during the first session was not due to an inherent perceptual limitation.

However, in contrast to a previous study on inattentional blindness in which every single participant was able to detect color desaturation after being informed about the manipulated phenomenon (Cohen et al., 2020), in our experiment, a significant number of participants still failed to correctly identify the critical period at the appropriate moment even after exposure to the manipulated stimuli. This disparity with Cohen et al.’s findings could be ascribed to the main task demand: Participants in the current study had to pay more attention to the primary task in a foveal window, which required sustained, high-load attention, whereas in Cohen et al. (2020), the participants were not actively engaged in an attentive task at the fovea. Supporting this interpretation, Cohen and colleagues demonstrated that participants were more likely to detect peripheral changes when engaged in a scene task requiring distributed attention across the visual field, compared to a face task requiring focused attention at the fovea (Cohen et al., 2021; Cohen & Rubenstein, 2020). These findings highlight the critical role of task demands in modulating inattentional blindness.

Another possibility is that our experiment involved a stronger inherent perceptual limitation than that in Cohen et al. (2020). Just as the detectability of peripheral degradation increases with the degree of degradation (Cohen et al., 2020; Cohen et al., 2021; Cohen & Rubenstein, 2020; Kim & Chong, 2024), the detectability of refresh-rate decrements in our study may have be influenced by modulating factors, such as the magnitude of the refresh-rate decrement and whether the transition is gradual or discrete. Therefore, it is possible that the refresh-rate decrement in our experiment was inherently less salient than the color desaturation used by Cohen et al. (2020), due to the specific values selected for those modulating factors. However, failure to detect the refresh-rate decrement in our study cannot be fully attributed to such perceptual subtlety, as most participants were able to distinguish the video with the changed refresh rate from the baseline video when answering the second question.

From the results of the second session, we deduce that the lack of awareness in the first session may not be attributable solely to memory decay (i.e., inattentional amnesia; Chen & Wyble, 2015; Wolfe, 1999). If memory decay were the primary factor, most participants would have correctly responded in the second session. Yet, many participants still did not. Therefore, the lack of awareness in the first session might be more closely linked to a lack of anticipation of the reduction in refresh rates, or a deficit in spatial attention, or the allocation of attentional resources toward the secondary window while engaged in the main task positioned in the foveal area.

The limited allocation of attention in digital environments suggests that people may not evenly distribute their attention across multiple windows and, therefore, may overlook events in areas outside of their main focus. These results align with previous research on peripheral visual awareness, where our perceptual experiences are known to be vulnerable to clutter (Rosenholtz, 2016; Zhaoping, 2023) and oblivious to significant changes (Cohen et al., 2020, Cohen et al., 2021; Cohen & Rubenstein, 2020; Henderson & Hollingworth, 1999; O’Regan et al., 1999). This limited peripheral awareness more likely stems from a limited capacity of attention (i.e., an information bottleneck) than from hardwired limitations in visual processing (e.g., retinal resolution) or memory decay (Kim & Chong, 2024).

In a similar vein, when navigating through displays, our useful field of view may not be wide enough to detect unusual visual events such as motion artifacts caused by low refresh rates in the periphery. While some earlier studies reported that motion detection remains effective in peripheral vision (McKee & Nakayama, 1984), it is possible that limited attention reduced the sensitivity of detecting motion juddering in this study. Supporting this possibility, several studies have demonstrated that endogenous spatial attention enhances temporal resolution (Hein et al., 2006; Sharp et al., 2018). Specifically, attention can bias the visual system toward shorter temporal integration windows or higher sampling rates, thereby improving the ability to segregate sequential visual inputs (Fiebelkorn & Kastner, 2019; Fiebelkorn et al., 2013; Fries, 2023; Wutz et al., 2018). Accordingly, in the present study, it is possible that reduced attention to the peripheral window impaired the temporal resolution, resulting in a failure to detect motion artifacts caused by decreased refresh rates.

Then, does the limitation of our attention in the periphery persist regardless of attentional fluctuations? According to our attentional state analysis, in the second session, we found that the detection of motion artifacts caused by decreased refresh rates was more frequently reported during out-of-the-zone than in-the-zone states. This tendency was consistent even when we excluded trials overlapping with the critical period, where the responses made during the trials could have influenced the response variability and led to out-of-the-zone states. In other words, this suggests that out-of-the-zone states might be more associated with processing task-irrelevant information or engaging in other cognitive processes than in-the-zone states and, correspondingly, implies that there is an advantage of being out-of-the-zone—that is, improved processing of distractors—compared to being in-the-zone. To this point, Decker et al. (2023) identified the advantages of being in an out-of-the-zone state, suggesting that such periods are not unproductive, as they may facilitate natural learning from less focused stimuli. In the out-of-the-zone states, the inhibition of spatially separated visual information might be less effective, thereby producing paradoxical benefits of enhanced distractor processing.

Although our main task was similar to and designed to mimic the experience of watching YouTube videos, it is important to acknowledge the unique characteristics of our main task, the gradCPT. In particular, it demands continuous focus on a display, a notably challenging task. Unlike other cognitive tasks, attentional or cognitive resources cannot be replenished after the end of short trials. While this setup allowed for high experimental control, it may limit ecological validity. However, our recent study (Yeo et al., 2024) also demonstrated that lowering the refresh rate of a task-irrelevant window did not impair video degradation detection in a task-relevant window. This suggests that our findings may be generalizable to more dynamic real-world contexts. An intriguing avenue for future research is examining how the degree of distraction caused by visual events in the periphery or a window located in the periphery varies depending on the cognitive load of the main task. As another possible limitation, our sample comprised only college students. However, because children (Plebanek & Sloutsky, 2017) and older adults (Amer & Hasher, 2014) use more distributed attention than young adults, they might notice unusual changes in the periphery more easily than this study’s participants did. Indeed, our participants were limited to young adults, and recent statistics indicate that this age group spends the most time on digital screens. Among individuals aged 16–24, women average 7 hours and 32 minutes of screen time per day, while men average 6 hours and 55 minutes. Among adults aged 25–34, women average 7 hours and 3 minutes, while men average 6 hours and 37 minutes (Freedom, 2024). This suggests that our sample represents a user group that is especially relevant to the context of screen-based technology usage. Accordingly, we suggest that future research examine how sensitivity to peripheral refresh-rate changes varies by age, particularly by comparing children, young adults and older adults.

We also acknowledge that we defined the in-the-zone and out-of-the-zone states based solely on RT variability. While attentional states can be assessed using a range of physiological and self-report measures (Smallwood & Schooler, 2006), our study focused on behavioral data collected during task performance. For this reason, we employed RT variability as an indirect behavioral index of attentional engagement. This approach is well established in the literature, particularly in the work of Esterman et al. (2013), who demonstrated that moment-to-moment fluctuations in RT variability can reflect dynamic changes in attentional state during sustained attention tasks.

RT variability has also been validated as a robust marker in clinical research. For instance, individuals with ADHD consistently exhibit elevated RT variability across diverse cognitive tasks, a finding that has been replicated in both adult and child populations (e.g., Epstein et al., 2011; Kofler et al., 2013). This suggests that RT variability captures meaningful individual differences in sustained attentional control. Additionally, as in prior studies, instead of relying on raw RT values alone, we applied Gaussian smoothing to account for temporal trends and minimize the influence of local noise. While we recognize that multimodal measures could offer a more comprehensive understanding of attentional engagement and immersion, we believe that, within the constraints of our behavioral paradigm, RT variability provides a theoretically grounded and empirically supported proxy for attentional fluctuations.

Overall, the current research investigates the sensitivity of peripheral perception when people explore the digital landscape, particularly concerning visual artifacts that the viewer may not be aware of. Our results suggest that variable-refresh-rate displays are a viable option for saving energy without diminishing user experiences. This study represents a crucial step forward in understanding human visual perception in a digital world given the extensive time spent using displays. Future studies can investigate whether these experiences differ for individuals who are adept at task switching, frequent multimedia users, those skilled in dividing their attention or those with a broader attentional span.

## Supplementary Information

Supplementary file 1.

## Data Availability

All raw data and MATLAB scripts for generating the stimuli and conducting the experiments have been made publicly available in the Open Science Framework (https://osf.io/3gcha). The data were analyzed using R (version 4.2.1) (R Core Team, 2020), and the figures were created using the ggplot2 package in R, version 3.4.0 (Wickham, 2016), and the seaborn package (version 0.11.2) in Python 3.8.6.

## Abbreviations

gradCPT: Gradual-onset continuous performance task
RT: Response time
VTC: Variance time course
BF: Bayes Factors
