# Supplementary material for: Effect of multi-refresh-rate method on user experience: sustained attention and inattentional blindness
Source: Cogn Res Princ Implic. 2025 Aug 15;10:50. doi: 10.1186/s41235-025-00663-0 (PMC12356812; doi:10.1186/s41235-025-00663-0)
Supplement: Supplementary file 1 [file 41235_2025_663_MOESM1_ESM.docx]

**Supplemental Materials**

**Effect of multi-refresh-rate method on user experience: sustained attention and inattentional blindness**

Supplementary Table 1: Descriptive Responses of the Participants after Question 1

| **20 Hz group** | **30 Hz group** |
| --- | --- |
| A thin light flickered towards the bottom of the screen. | The trajectory of the rotating red circle became distorted. My attention kept drifting, and the gaze dropped down, resulting in a few incorrect presses. |
| There was a slight feeling that the screen moved up to the top right in the middle. | The circle moved up and down. |
| The ball slowed down then sped up (rotation speed). | It seemed like the rotating white-big circle got thicker. |
| The image was presented with a slight time delay, causing it to appear overlapped and fused. | It seemed like the direction of the rotating red dot changed. |
